# Design and Test of a New Dielectric-Loaded Resonator for the Accurate Characterization of Conductive and Dielectric Materials

**DOI:** 10.3390/s23010518

**Published:** 2023-01-03

**Authors:** Andrea Alimenti, Kostiantyn Torokhtii, Pablo Vidal García, Nicola Pompeo, Enrico Silva

**Affiliations:** 1 Department of Industrial, Electronic and Mechanical Engineering, Roma Tre University, Via Vito Volterra 62, 00146 Roma, Italy; 2 Istituto Nazionale di Fisica Nucleare INFN, Sezione Roma Tre, Via della Vasca Navale 84, 00146 Roma, Italy

**Keywords:** dielectric-loaded resonator, surface resistance measurement, complex permittivity measurement, microwave material characterization, 3D printing materials

## Abstract

The spread of additive manufacturing techniques in the prototyping and realization of high-frequency applications renewed the interest in the characterization of the electromagnetic properties of both dielectric and conductive materials, as well as the design of new versatile measurement techniques. In this framework, a new configuration of a dielectric-loaded resonator is presented. Its optimization, realization, and use are presented. A measurement repeatability of about one order of magnitude lower than the commonly found values ($10^{-3}$ on the *Q*-factor and $15\times 10^{-6}$ on the resonance frequency, given in terms of the relative standard deviations of repeated measurements) was reached thanks to the design of a closed resonator in which the samples can be loaded without disassembling the whole measurement fixture. The uncertainty levels, the ease of use, and the versatility of the realized system make its use of potential interest in numerous scenarios.

## 1. Introduction

The characterization at microwave frequencies of the electromagnetic (e.m.) properties of materials has always been a field of great interest due to the relevance that these measurements can have in the design and performances of telecommunication systems. For this reason, a large number of different high-frequency characterization techniques have started to appear in the literature since the 1950s. Several techniques were designed to meet the different measurement needs in terms of the operative band, accuracy, and characteristics of the material under investigation [1,2,3,4,5,6,7,8,9,10,11,12].

In recent years, new materials and manufacturing techniques, of interest also for telecommunication and sensing applications, have emerged. For example, the possibility of printing dielectric structures either with complex shapes or with geometrically controlled e.m. properties was investigated for the realization of radar sensors, antennas, graded-index lenses, etc. [13,14,15,16]. Moreover, printed conductive materials were under study for high-frequency flexible sensing applications, guiding structures, antennas, etc. [17,18,19]. For these reasons, in recent years, the interest in the e.m. characterization of conductive and dielectric materials was revived.

In this work, we describe the design and test of a new application of the Hakki–Coleman configuration of a dielectric-loaded resonator useful for the characterization of both dielectrics and conductors. Thus, the physical quantities of interest in this study are the complex relative permittivity $\tilde{\varepsilon }=\varepsilon^{\prime }-i\varepsilon^{\prime \prime }$ [20] for dielectrics and the surface resistance $R_{s}$ [20] for conductors. The here presented measurement fixture was designed with the aim of obtaining a versatile system for laboratories committed to the prototyping of high-frequency systems also through the use of additive manufacturing techniques. For this reason, the three main design constraints were (i) the need to obtain a system able to work both with conductors and dielectrics, (ii) the ease of use, and (iii) a target uncertainty of ∼1 mΩ for $R_{s}$ measurements on normal conductors and <5% on the real and imaginary part of $\tilde{\varepsilon }$.

Due to the required accuracy level, a resonant system was selected on purpose. In particular, the dielectric-loaded resonator (DR) fixture was chosen for its high sensitivity, which is in fact generally exploited, and also for the characterization of low-loss materials and even superconductors [2,21,22,23,24,25,26,27,28,29,30,31,32]. However, due to their high sensitivity, microwave resonant techniques generally suffer from a low measurement repeatability, being particularly sensitive to small variations in the sample mounting. For this reason, we designed a DR which does not require a complete disassembling for each measurement: the idea was to produce a closed DR on which the samples under investigation can be mounted from the outside thanks to the presence of windows on the cavity and the dedicated sample holders. In this way, in addition to improving the mounting repeatability, it was possible to obtain an easy-to-use fixture even for non-expert users and to fasten the measurement procedure with respect to other common approaches. Finally, for what concerns the need of characterizing both conductors and dielectrics, the resonator geometry and the sample holders were optimized to guarantee the best sensitivity on $R_{s}$ and $\tilde{\varepsilon }$ considering the properties of the materials of interest.

The paper is organized as follows: In Section 2, the measurement method is introduced; then the design and geometry optimization and realization of the measurement fixture are shown in Section 3; and finally, the experimental use of the realized DR on the $R_{s}$ and $\tilde{\varepsilon }$ measurements is shown in Section 4. A brief summary of the work is provided in Section 6.

## 2. Measurement Methods

**Figure 1:**
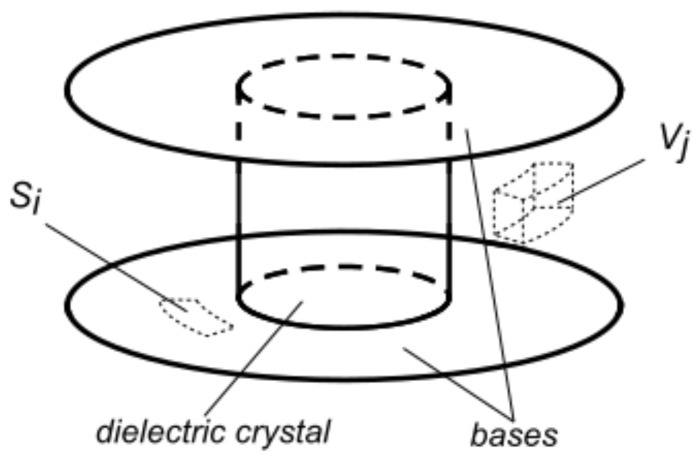
Sketch of a cylindrical dielectric-loaded resonator (DR). Between the metallic bases, a low losses dielectric crystal is loaded to increase the quality factor *Q*, and thus the sensitivity, of the DR. When used as a fixture for the measurement of the surface resistance $R_{s}$ of conductors, a part of the surface $S_{i}$ of the metallic enclosure is substituted with the sample under study while, when used for the characterization of dielectric materials, the sample of volume $V_{j}$ is loaded in the DR.

The used technique is based on the measurement of the quality factor *Q* and the resonance frequency $f_{0}$ of a dielectric-loaded resonator (see Figure 1 for a sketch of the structure). Because *Q* and $f_{0}$ depend on the e.m. properties and geometries of every component of the resonator, then the insertion of a sample under test into the resonator will cause a change in *Q* and $f_{0}$. From the change in these quantities, the e.m. properties of the sample can be, at least in principle, obtained [2].

In particular, it can be shown that the *Q* factor of a resonator is determined by the components of the resonator itself as follows [2]:

(1) $$\frac{1}{Q}=\sum_{i}\frac{R_{s,i}\int_{S_{i}}{\left|H_{\tau }\right|}^{2}dS_{i}}{4\pi f_{0}W}+\sum_{j}\frac{\varepsilon_{0}\varepsilon_{j}^{\prime }\int_{V_{j}}{\left|E\right|}^{2}dV_{j}}{2W}\tan\delta_{j}:=\sum_{i}\frac{R_{s,i}}{G_{i}}+\sum_{j}\eta_{j}\tan\delta_{j}\;,$$

where *W* is the energy stored in the DR at the resonance and $H_{\tau }$ is the magnetic field that is tangential to the *i*-th conductive surface $S_{i}$ with surface resistance $R_{s,i}$, in the DR. Meanwhile, *E* is the electric field in the *j*-th dielectric element of volume $V_{j}$ and loss tangent $\tan\delta_{j}:=\varepsilon_{j}^{\prime \prime }/\varepsilon_{j}^{\prime }$.

Thus, from a measurement of *Q*, the measurements of $R_{s}$ or $\tan\delta_{s}$ of a sample under study could be obtained once the $R_{s,i}$, $G_{i}$, $\tan\delta_{j}$ and $\eta_{j}$ of all the other components of the resonator are known. This would require a complete calibration of the resonator, also taking into account the dependencies of these quantities on all the possible variables of influence (e.g., temperature, pressure, humidity). Because this procedure generally provides large measurement uncertainties, a different strategy, based on a perturbation method, is usually preferred.

Because different measurement procedures are followed to obtain the quantities of interest, i.e., $\tilde{\varepsilon }$ or $R_{s}$, the measurement methods used for the two classes of materials are described separately in the next subsections.

### 2.1. Surface Resistance $R_{s}$ Measurement Method

The end-wall perturbation method [2] is used to obtain the variation in $R_{s}$ of the material under investigation with respect to a reference sample of surface resistance $R_{s,ref}$:

(2) $$R_{s}-R_{s,ref}=G_{s}\left(\frac{1}{Q}-\frac{1}{Q_{ref}}\right)\;,$$

where $Q_{ref}$ is the quality factor measured once the reference sample is loaded in the DR. Equation (2) is obtained from Equation (1), assuming that during the two measurements (i.e., that with the sample of unknown $R_{s}$ and that with the reference one $R_{s,ref}$) the change in all the other quantities can be neglected.

The resonance frequency can be used to measure the variations in the surface reactance $X_{s}$ of the material as follows:

(3) $$X_{s}-X_{s,ref}=-2G_{s}\frac{f_{0}-f_{0,ref}}{f_{0,ref}}\;.$$

Actually, in conventional conductive materials, at the frequency of interest, $X_{s}=R_{s}$ [20,33]; thus, once $R_{s}$ is determined from *Q*, there is no further need for using $f_{0}$ measurements. The use of *Q* instead of $f_{0}$ for $R_{s}$ measurements is motivated by the different sensitivities of *Q* and $f_{0}$ to small variations both in the mounting and positioning of the sample and in influence quantities, such as temperature and pressure. The higher sensitivity of $f_{0}$ to all these phenomena generally makes *Q* measurements more reliable.

The identification of a reliable reference sample goes beyond the scope of this work. Only in principle $R_{s}=\sqrt{\mu_{0}\pi f\rho }$, with ρ the dc resistivity of the material. Just as a counter example, it is well known from the literature that the surface roughness $R_{g}$ strongly affects $R_{s}$ [34,35,36,37], but only empirical models $R_{s}\left(R_{g}\right)$ exist [38,39,40]. Thus, the simple derivation of $R_{s}$ from ρ measurements cannot be considered reliable for the realization of a measurement standard. For this reason, in this work, the metrological performances—in terms of measurement precision and accuracy—of the designed DR will be evaluated on $\Delta R_{s}$ measurements and not on absolute $R_{s}$. However, a procedure for the evaluation of the absolute $R_{s}$, without the need for a reference, will be presented at the end.

### 2.2. Complex Permittivity $\tilde{\varepsilon }$ Measurement Method

In this case, because two independent quantities (i.e., $\varepsilon^{\prime }$ and $\varepsilon^{\prime \prime }$) must be measured, both *Q* and $f_{0}$ of the DR are exploited.

The volume perturbation method is used [2]: a part of the inner volume of the DR is substituted with the sample under investigation and then, in the very same volume, the reference dielectric material is loaded. From the variations in *Q* and $f_{0}$ measured in the different configurations, $\tilde{\varepsilon }$ can be obtained. The resonance frequency $f_{0}$ can be used to measure $\varepsilon^{\prime }$, while *Q*, as also shown in Equation (1), is used to obtain tanδ and thus $\varepsilon^{\prime \prime }$, once $\varepsilon^{\prime }$ is known. In general, measurements of $f_{0}$ with respect to the reference are linked to $\varepsilon^{\prime }$ by

(4) $$\varepsilon^{\prime }=g_{1}\left(\frac{f_{0}-f_{0,ref}}{f_{0,ref}}\right)\;,$$

where $g_{1}$ is a calibration curve that depends on the geometry, the position of the sample in the DR, and on $\varepsilon_{ref}^{\prime }$. The calibration function $g_{1}$ can be obtained through e.m. simulations of the DR.

Once $\varepsilon^{\prime }$ is obtained, the filling factor can also be determined using the calibration curve $\eta_{s}=g_{2}\left(\varepsilon^{\prime }\right)$, similarly obtained by simulations. With $\eta_{s}$, Equation (1) can be computed to obtain tanδ, which in the small perturbation limit can be reduced to

(5) $$\tan\delta_{s}\approx \frac{1}{\eta_{s}}\left(\frac{1}{Q}-\frac{1}{Q_{ref}}-\eta_{s}\tan\delta_{ref}\right)\;.$$

When the small perturbation limit is no longer valid, Equation (1) as a whole must be used, taking into account the variation in $G_{i}$ and $\eta_{j}$ caused by the insertion of a sample with a different $\varepsilon^{\prime }$ with respect to the reference.

A very practical reference can be the air itself: within the typical measurement uncertainties, one can assume $\varepsilon_{ref}^{\prime }\approx 1$ and $\tan\delta_{ref}\approx 0$ without introducing significant errors.

## 3. Design and Realization of the DR

The DR was designed starting from these requirements:

1. Improvement in the measurement repeatability: a closed configuration in which the sample can be loaded from the outside without the need of disassembling the whole DR for each measurement is preferred;
2. Possibility of hosting two samples at the same time: this can be used to perform multiple-sample comparisons [41] or to increase the sensitivity when needed;
3. Contactless measurements: the sample holder must be designed to support the samples without letting the probed area of the sample touch other surfaces. This is useful to avoid damaging delicate sample surfaces and/or coatings.

**Figure 2:**
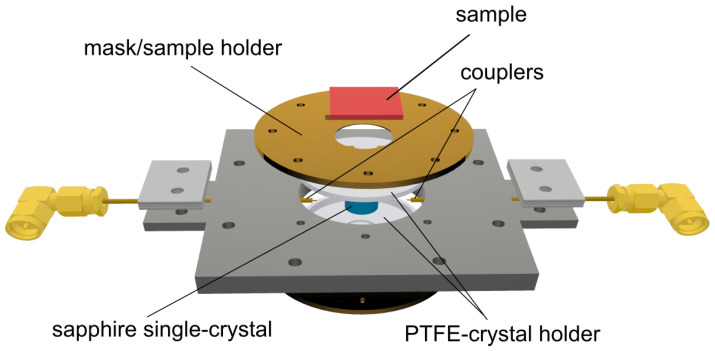
Three-dimensional exploded view of the DR design.

To meet these requirements, a closed structure was designed with open windows on the resonator bases from which the samples can be exposed to the resonator inner volume, as shown in Figure 2. In order to keep the dielectric crystal in position, despite the openings on both bases, a polytetrafluoroethylene (PTFE) holder, pressed into the metallic cavity, was used. The PTFE was chosen because of its good rigidity and relatively low $\varepsilon^{\prime }$ and tanδ. In order to reduce the detrimental impact of the PTFE holder on the *Q*-factor of the DR, its geometry was carefully designed to obtain a small filling factor: the volume occupied by the holder is far from peaks of the *E* field.

The bases of the resonator were designed to fill the dual role of the sample holder and mask: a rectangular housing was performed to host and center the samples from the outside of the DR. In its center, a circular hole allowed the e.m. mode to probe the surface of the sample without disturbing the mode symmetry. This also allowed the contactless mounting of the sample: in fact, the surface of the sample facing the central hole was not in contact with any surface.

Finally, the resonator was excited in the TE_011_ mode through coaxial cables (see Figure 2) ended with magnetic loops. The TE_011_ mode was chosen because, due to the field configuration, it was not necessary to ensure a perfect electrical contact between the bases and the lateral wall. In addition to this, the circular symmetry implies that no electrical contact was needed between the sample and the mask. Moreover, the TE_011_ is usually well separated from other spurious modes, helping its recognition and avoiding disturbances of near modes.

### 3.1. Dimensions Optimization—$R_{s}$ Measurements

Once the DR structure was chosen, the optimization of its dimensions to maximize the measurement sensitivity of typical conductive samples was performed through e.m. simulations. We simulated a full 3D structure of the resonant cell, exploiting the Finite Element Method (FEM) with the eigenmode Comsol solver. Because the design of the structure required the optimization of a large set of parameters, we developed a custom external script to automatically identify the TE_011_ mode-related parameters. The automatically adaptive finer mesh was used for all the structures and an extra fine mesh was used for the dielectric and mask parts.

In particular, the crystal dimensions, the PTFE thickness, and air gaps between the crystal and the samples have to be carefully chosen. The radius of the wall of the metallic enclosure of the DR was simply chosen large enough to make negligible the conduction losses on the lateral wall itself while avoiding other cavity modes close to the TE_011_. Thus, the cavity radius was fixed at $R_{cav}=15$ mm.

The DR measurement sensitivity $c_{R}$ to the sample surface resistance $R_{s}$ is the parameter to maximize: from Equation (1), $c_{R}=\left|\partial Q/\partial R_{s}\right|=Q^{2}/G_{s}$. The optimization was performed by varying the heights of the gaps and analyzing $c_{R}$ given by one available sapphire single-crystal cylinder of dimensions ∅=8.00(1) mm and height h=5.00(1) mm. The simulations were performed setting R=60mΩ for all the metallic (brass) surfaces as a realistic surface resistance value [42].

**Figure 3:**
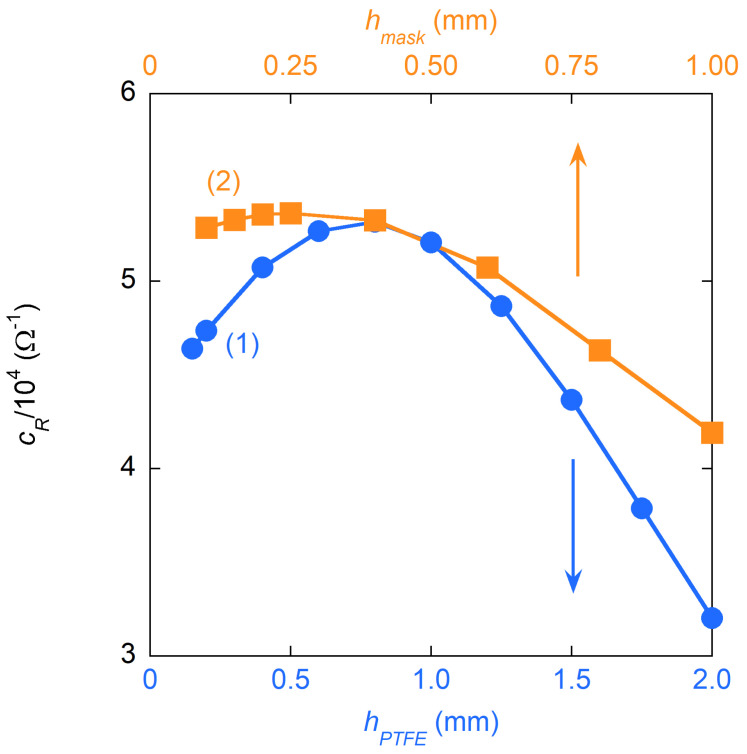
The measurement sensitivity is maximized through the optimization of the geometry of the resonator. Once the dimensions of the dielectric crystal are fixed, the thickness $h_{PTFE}$ of the PTFE layer between the crystal and the sample holder, and that of the metallic mask $h_{mask}$, can be optimized. The optimization is performed with e.m. simulations by exploring the effects on the sensitivity coefficient $c_{R}=Q^{2}/G_{s}$ when $h_{PTFE}$ and $h_{mask}$ are varied within mechanically reasonable limits. Curve 1 (blue circles): sensitivity $c_{R}$ optimization by varying $h_{PTFE}$ (lower horizontal axis). Curve 2 (orange squares): sensitivity $c_{R}$ optimization by varying $h_{mask}$ (upper horizontal axis) once fixed $h_{PTFE}=0.75$ mm, which corresponds to the maximum *c* value shown in curve 1.

The optimization of the thickness $h_{PTFE}$ of the PTFE holder between the bases and the sapphire crystal was performed by simulating the $c_{R}$ of the DR, changing $h_{PTFE}$ in the interval $0<h_{PTFE}/\mathrm{mm}<2$, as shown in curve 1 in Figure 3. A maximum, at $h_{PTFE}∼0.75$ mm, exists in $c_{R}\left(h_{PTFE},h_{mask}\right)$ because of the different sensitivity of *Q* and $G_{s}$ to $h_{PTFE}$. With this optimum $h_{PTFE}$ value, the effect of $h_{mask}$ on $c_{R}$ was also studied (see curve 2 in Figure 3). It was chosen $h_{mask}=0.3$ mm as the best compromise between the maximum $c_{R}$ and the minimum thickness reachable to guarantee a sufficiently rigid brass surface usable for the sample holder.

### 3.2. Dimensions Optimization—$\tilde{\varepsilon }$ Measurements

Once the DR was optimized for $R_{s}$ measurements, the best dimensions of the measurable dielectric samples, and thus of the sample holder, were assessed from the point of view of the sensitivity on the $\tan\delta_{s}$ measurement. The sensitivity coefficient is obtained from Equation (1) as follows:

(6) $$c_{\tan\delta }=\frac{\partial Q}{\partial \tan\delta_{s}}=-\frac{\eta_{s}}{{\left(\sum_{i}\frac{R_{s,i}}{G_{i}}+\sum_{j}\eta_{j}\tan\delta_{j}\right)}^{2}}=-\eta_{s}Q^{2}\;.$$

Then, to maximize the sensitivity $c_{\tan\delta }$, $\eta_{s}$ must be optimized depending on the $\tan\delta_{s}$ of the sample. Thus, a study of $c_{\tan\delta }\left(\tan\delta_{s},\eta_{s}\right)$ must be performed.

Defining the quantity $b_{r}:=Q^{-1}-\eta_{s}\tan\delta_{s}$, i.e., a measure of the overall losses apart from those due to the sample, it is possible to identify two different asymptotic limits for $c_{\tan\delta }$, one in which $\eta_{s}\tan\delta_{s}\gg b_{r}$ and the second for $\eta_{s}\tan\delta_{s}\ll b_{r}$. In the first case, one obtains $|c_{\tan\delta }|\to \eta_{s}^{-1}{\left(\tan\delta_{s}\right)}^{-2}$, while in the second $|c_{\tan\delta }|\to \eta_{s}b_{r}^{-2}$ which, as expected, is no longer dependent on $\tan\delta_{s}$. In other words, when the losses in the dielectric sample are large with respect to the other losses into the resonator, then the measurement sensitivity decreases due to the lowering of the *Q* factor: in this case, a smaller sample is useful to reduce $\eta_{s}$. Whereas, when the losses in the sample are smaller than the others, then the sensitivity of the resonator is limited by its intrinsic *Q*: in this case, a bigger sample is useful to increase the amount of losses in the sample. Hence, the best $\eta_{s,opt}$ for a fixed $\tan\delta_{s}$ is obtained at the crossover of these two opposite scenarios, that is, when the losses in the sample are the same as those in the cavity, $\eta_{s,opt}\tan\delta_{s}/b_{r}=1$, which can be analytically obtained from Equation (6). The corresponding maximum sensitivity is $|c_{\tan\delta }{|}_{max}=1/\left(4b_{r}\tan\delta_{s}\right)$.

Thus, the best dimension of the sample can be evaluated starting from $b_{r}$ and a rough estimation of $\tan\delta_{s}$. From these, the optimum filling factor is evaluated $\eta_{s,opt}=b_{r}/\tan\delta_{s}$. Finally, through e.m. simulators, the dimensions of the sample can be assessed in order to obtain $\eta_{s,opt}$ using an expected value for $\varepsilon^{\prime }$. Hence, there is not an optimum configuration for every kind of dielectric sample, but the design of the DR should remain versatile enough to accommodate samples of different dimensions. This means that a set of different sample holders can be designed and realized in order to accommodate samples with different e.m. properties, and thus to always exploit the maximum measurement sensitivity of the resonator. In particular, sample holders with holes of different diameters can be useful for this application. In addition to this, it is also possible to act on the thickness of the samples when they are specifically prepared for characterization.

### 3.3. Realization of the DR

**Figure 4:**
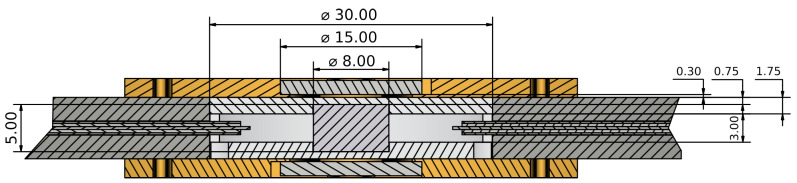
Vertical straight section of the designed DR. All the dimensions are in mm.

**Figure 5:**
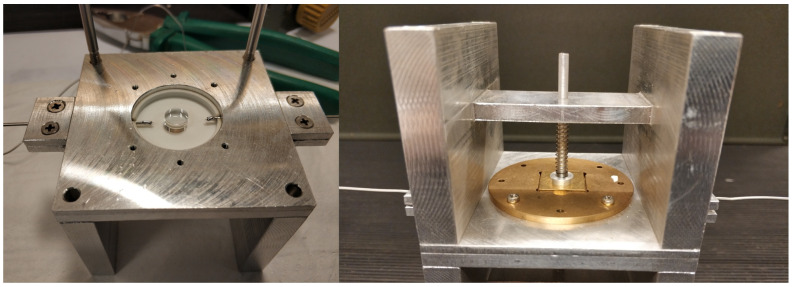
Pictures of the realized DR. On the left, the open cavity with the sapphire crystal. On the right, the closed DR with the external supporting structure.

The so-optimized design was realized by also taking into account constraints regarding the cost, hardness, and minimum workable dimensions of the different materials. The final dimensions of the optimized configuration are reported in Figure 4 and pictures of the realized DR are shown in Figure 5.

## 4. Experimental Tests and Performances Analysis

In this section, the metrological characteristics of the realized resonator are first experimentally assessed, and then the examples of the use of the DR for the characterization of both the conductive and dielectric materials are provided.

The transmission and reflection scattering S-parameters are measured with an Anritsu 37269D Vector Network Analyzer (VNA), setting the center frequency on the peak of the resonance, a span of ∼5 times the full-width half-maximum (FWHM) (as shown in [43,44,45] for an improved measurement accuracy) and 1601 evenly spaced points (the maximum allowed number of points for a frequency sweep). The emitted power is set at −10 dBm and the Intermediate Filter (IF) bandwidth at 10 kHz. The DR is linked to the VNA with 60 cm long phase-stable semi-rigid K coaxial cables, Anritsu 3670KF50-2. The transmission line is calibrated with the standard Short-Open-Load-Through (SOLT) calibration procedure before performing the measurement. At this calibration plane, the DR is connected through 10 cm semi-rigid K coaxial cables ended with the couplers into the DR cavity.

From the transmission scattering parameters, the loaded $Q_{l}$ and $f_{0}$ are evaluated, fitting the resonance curves with the following modified Lorentzian model [46,47,48]:

(7) $$\left(\frac{A}{1+i2Q_{l}\left(\frac{f-f_{0}}{f_{0}}\right)}+B\right)e^{i(\alpha +f\beta )}\;,$$

where *B* is a complex constant representing the cross-coupling between the two ports, *A* the peak of the unperturbed Lorentzian curve, and α+fβ the phase delay given by the uncalibrated final part of the transmission line.

The coupling is set to be so low to allow the approximation $Q\approx Q_{l}$. In the case of non-negligible coupling, the unloaded *Q* is evaluated through the TMQF algorithm [11,49].

**Figure 6:**
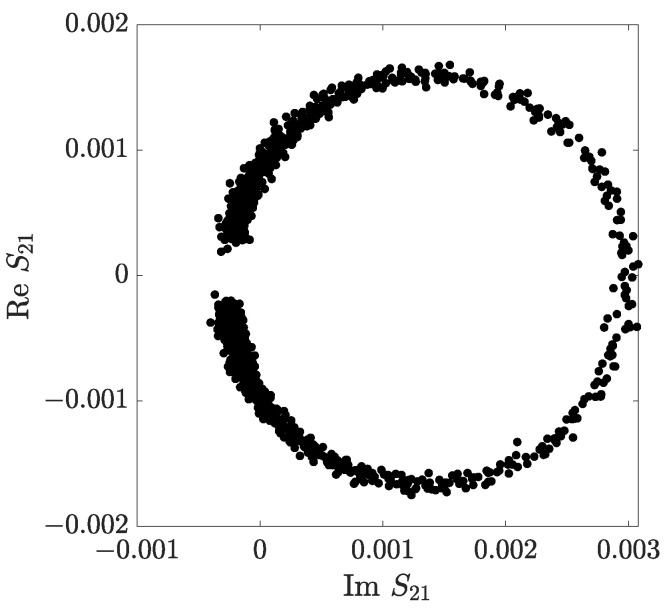
Measurement of the S_12_ parameter on the complex plane.

An example of the measurement of the transmission S_21_ parameter around the resonance frequency is shown in Figure 6. The position of the out-of-resonance points shows the small cross-coupling of the DR, B≈−0.00026+i0.00002.

### 4.1. Measurement Repeatability

Due to the typical high sensitivity of the DR on the mounting, the measurement repeatability performances of the DR were tested by repeating measurements disassembling and re-mounting a conductive sample each time. The repeatability test was performed in the single-sample configuration (closing the lower base with the brass cap) and using a copper sample of nominal dimensions $15\times 15\times 3\;{\mathrm{mm}}^{3}$. A mass of ∼200 g was placed on the sample to improve the repeatability.

**Figure 7:**
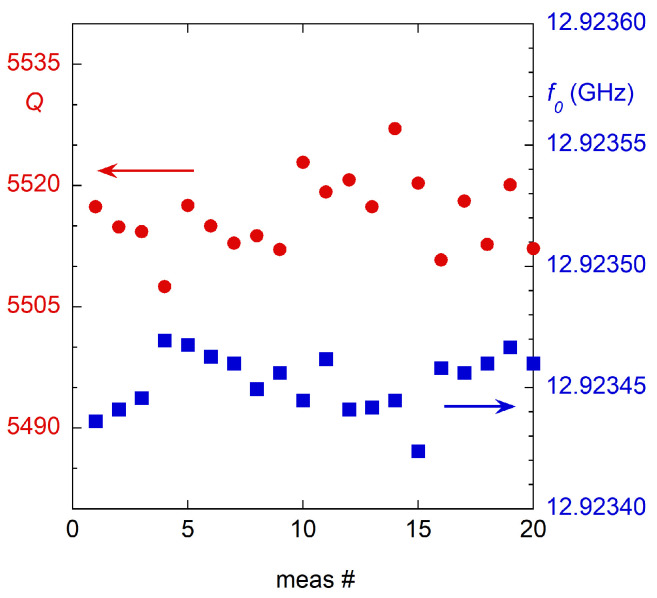
*Q* (red circles) and $f_{0}$ (blue squares) measurement repeatability evaluation.

In Figure 7, the *Q* and $f_{0}$ measurement repetitions are shown. With this new DR design, it was possible to reduce the *Q* mounting repeatability (evaluated as the standard deviation of the experimental points) from a typical 5% [25] to 0.1%. Meanwhile, the resonance frequency repeatability is here assessed to be ∼$15\times 10^{-6}$. These are the largest contributions on u(Q) and $u(f_{0})$. In fact, the uncertainties u(Q) and $u(f_{0})$ evaluated by the fitting algorithm from the residuals are more than an order of magnitude lower than those provided by the mounting repeatability. In addition to this, u(Q) and $u(f_{0})$ obtained by the fitting procedure can be easily reduced by narrowing the IF bandwidth or performing an average on the acquisition of the single points in the frequency, whereas the contributions of the mounting are not improved by this. Thus, in other words, the mounting repeatability is an intrinsic characteristic of the designed measurement fixture, whereas the other contributions on u(Q) and $u(f_{0})$ depend on the measurement procedure and instrumentation used.

### 4.2. $R_{s}$ Measurements and Uncertainty Evaluation

The performances of the designed resonator on the measurements of $R_{s}$ are here reported. However, because the measurement of the absolute $R_{s}$ requires a complete calibration of the resonator, an accurate measurement of all $R_{s,i}$ and $\tan\delta_{j}$ (see Equation (1)) is necessary, in addition to the accurate determination of the geometrical and filling factors. For this reason, to highlight the proper metrological characteristics of the DR to $R_{s}$ measurements, we first show the application to differential $\Delta R_{s}$ measurements and then to absolute $R_{s}$ measurements. The uncertainty affecting this last type of measurement is in fact largely determined by the uncertainties on the e.m. properties of the components of the resonator: these could improve with the development of novel measurement techniques, and they are not determined by the design of the here presented measurement fixture.

In the next sections, the measurement performances of the realized DR will be experimentally tested both on the ΔR and *R* measurements. The samples used for this evaluation are reported in Table 1.

#### 4.2.1. Differential $\Delta R_{s}$ Measurement

In several cases, one is not interested in the absolute $R_{s}$ value but instead in the differences $\Delta R_{s}$ between the samples in order to evaluate, for example, which surface treatment gives the best results. In this case, no calibration of the resonator is needed so that the uncertainties $u(\Delta R_{s})$ can be very small.

To lighten the mathematical notation, we define the sum of the whole dielectric losses as $l_{d}:=\sum_{j}\eta_{j}\tan\delta_{j}$, the inverse of the unloaded quality factor as $l:=Q^{-1}$, and that of the geometrical factors as $A_{i}:=G_{i}^{-1}$.

Because with differential $\Delta R_{s}$ measurements $l_{d}$ and the geometrical factors can be assumed constant among the different measurements (if the difference $\Delta R_{s}$ in the samples is not so large to change the field configuration and negligible variations in the measurement influence quantities are present), one obtains:

(8) $$\Delta R_{s}=R_{s,i}-R_{s,ii}=\frac{l_{i}-l_{ii}}{A_{s}}\;,$$

where $l_{i}$ and $l_{ii}$ are the inverses of the quality factors measured when the samples with surface resistance $R_{s,i}$ and $R_{s,ii}$ are loaded.

The measurements are performed with a brass mask with $\emptyset_{1}=13.00\left(1\right)$ mm with a geometrical factor $G_{1}=10.2\left(1\right)$ kΩ. From this, the geometrical factor of the sample is evaluated, obtaining $G_{s1}=1.31\left(1\right)$ kΩ. The number in parentheses is the numerical value of the standard uncertainty u(G) referred to the corresponding last digits of the quoted parameter. u(G) are evaluated by the e.m. simulations, taking into account the uncertainties on the physical dimensions of the resonator $u\left(h_{cav}\right)/h_{cav}=0.2\%$, where $h_{cav}$ is the height of the cavity, the uncertainties on the dielectric crystal relative permittivity ($9<\varepsilon^{\prime }<10$), and the variation in the effective length of the cavity given by the field penetration length in Cu at 13 GHz ($\delta_{Cu,13\mathrm{GHz}}∼0.6$ μm). The contribution to the geometrical factors uncertainties given by these three effects are, respectively, $u{\left(G\right)}_{dim}/G∼0.5\%$, $u{\left(G\right)}_{\varepsilon^{\prime }}/G∼1\%$, and $u{\left(G\right)}_{\delta Cu,16\mathrm{GHz}}/G∼0.3\%$. Thus, the main contribution is given by $u(\varepsilon^{\prime })$ of the sapphire crystal. The uncertainties on the physical dimensions of the cavity and of the mask are the mechanical tolerances of the numerical control tool used for manufacturing.

Taking into account the type-A evaluation of the measurement uncertainty reported in Section 4.1, the obtained $\Delta R_{s}$ measurements are shown in Table 2:

As expected, the uncertainties are “small” in this case because those are determined only by u(Q) and $u(G_{i})$. Once again, this highlights the importance an $R_{s}$ measurement standard, currently missing, would have in microwave measurements. Thanks to its high measurement repeatability, the DR here presented allows discriminating samples with ΔR≥2mΩ which is a sufficiently accurate measurement for standard microwave applications.

#### 4.2.2. Absolute $R_{s}$ Measurement

Because it is well known that the actual $R_{s}$ of metallic samples is often far from the nominal $R_{s}$ value evaluated from the dc resistivity ρ [50,51], an accurate calibration cannot rely on an analytically computed $R_{s}$. Moreover, for $l_{d}$, the use of the literature values is not recommended due to the large dispersion of the tanδ values associated with sapphire and different kinds of PTFE. For these reasons, an in situ calibration procedure was developed, exploiting the properties of the new DR.

The aim is to find a combination of independent measurements that allows the determination of $l_{d}$ and the surface resistance $R_{0}$ of the brass bases of the resonator (the contribution of the aluminum lateral wall on the *Q* is negligible in this configuration), once the geometrical factors are estimated by numerical simulations (indeed, there is no way to measure geometrical factors without a calibrated standard [25]). The minimum set of independent measurements is obtained by performing the first two measurements using two pairs of samples of different materials (one of them of the same material as the cavity $R_{0}$) and performing the third measurement by changing the DR geometrical factors. In this way, there is no need to use a third unknown material, and $l_{d}$ can be determined. The geometrical factors of the resonator can be changed using masks with different diameters of the central hole, through which the samples are exposed to the DR.

Mask $M_{i}$ (i=1,2) has a hole diameter $\emptyset_{i}$, a geometrical factor $G_{i}=A_{i}^{-1}$, and exposes a sample area of the geometrical factor $G_{si}=A_{si}$. Due to the structure symmetry, the bases of the resonator are considered equivalent. The set of measurements is represented by the following system:

(9) $$\left(\begin{array}{ccc} 2A_{s1} & 2A_{1} & 1\\ 0 & 2A_{s1}+2A_{1} & 1\\ A_{s2} & A_{s1}+A_{1}+A_{2} & 1 \end{array}\right)\;\cdot \;\left(\begin{array}{c} R_{x}\\ R_{0}\\ l_{d} \end{array}\right)=\left(\begin{array}{c} l_{1}\\ l_{2}\\ l_{3} \end{array}\right)\;,$$

where $R_{x}$ is the surface resistance of an unknown sample and the conduction losses of the lateral wall of the resonator are neglected. The system (9) allows to find $R_{0}$, $l_{d}$, and $R_{x}$.

The following calibration procedure was used with two pairs of brass and copper samples. The masks are entirely made of brass and the DR is used in the dual sample configuration (i.e., with samples mounted on both bases). One of these is that used in the previous measurement (see Section 4.2.2 for the dimensions), while the second one has the central hole of diameter $\emptyset_{2}=9.00\left(1\right)$ mm and a geometrical factor $G_{2}=2.84\left(3\right)$ kΩ. The samples’ geometrical factors are then $G_{s1}=1.31\left(1\right)$ kΩ (with the first mask) and $G_{s2}=1.96\left(2\right)$ kΩ (with the second mask).

By solving system (9), the measured volume losses are $l_{d}=4\left(2\right)\times 10^{-5}$ and $R_{0}=92\left(12\right)$ mΩ. Assuming that the sapphire crystal filling factor is ∼1 (in dielectric-loaded resonators, thanks to the high dielectric permittivity $\varepsilon^{\prime }$ of the crystal used, the electromagnetic field is confined in the volume of the crystal itself; thus, the energy stored in the resonator is in the first approximation that is contained in the dielectric crystal [52]), then $l_{d}∼\tan\delta_{sap}$. The obtained $\tan\delta_{sap}$ of the sapphire crystal is well in agreement with the room temperature value reported in [53]. Moreover, $R_{0}$ is compatible with that of brass, considering that the surface roughness can double [38] the nominal *R* evaluated from the dc resistivity of the material (see Table 1).

The DR is then tested with the samples in Table 1. The results obtained with the single-sample configuration are reported in Table 3.

The large uncertainties are given in this case by the large uncertainty on $l_{d}$. It must be noticed that the measured *R* seems to be particularly far from the ideal values shown in Table 1. This evident discrepancy can be interpreted according to the empirical models [54] that describe the dependence of *R* on the surface roughness $R_{g}$ of the sample. $R_{g}$ is estimated to be in the range (0.9÷3.0) μm through these same models which is a roughness level well compatible with what is expected on the used samples.

### 4.3. $\tilde{\varepsilon }$ Measurements and Uncertainty Evaluation

The versatility of the designed measurement fixture allows its use both for the $R_{s}$ measurements of conductive samples and for the $\tilde{\varepsilon }$ of dielectric materials. In this section, we show an example of the use of the designed DR for the characterization of dielectric materials.

**Figure 8:**
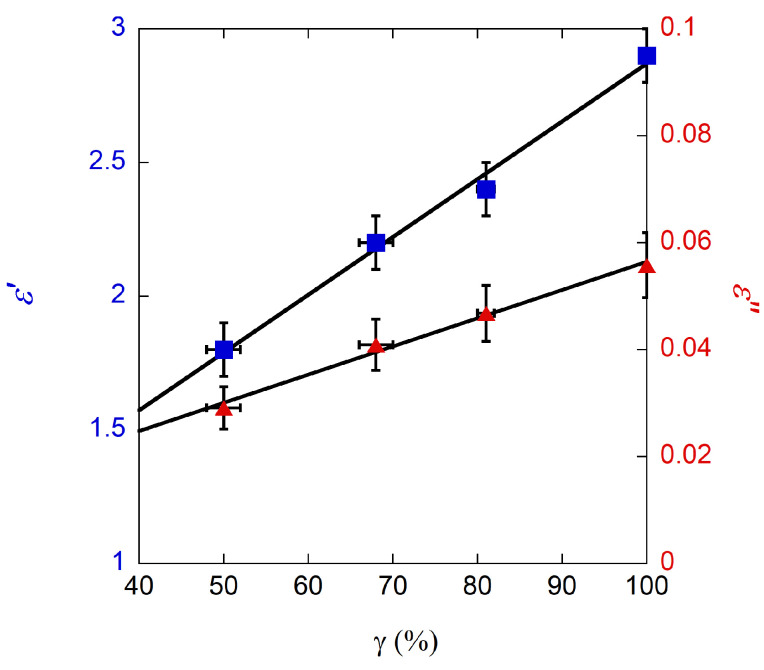
Real part of the relative complex permittivity (blue circles, left axis) and imaginary part (red triangles, right axis) measured on samples prepared with different porosity. The horizontal axis shows the filling percentage γ of the samples.

**Figure 9:**
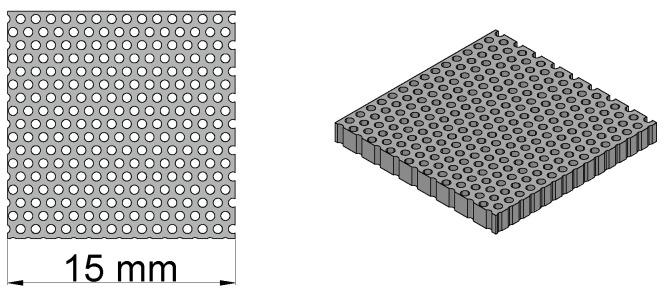
Three-dimensional drawing of the printed sample at 68% filling.

In order to experimentally test the DR in a wide $\tilde{\varepsilon }$ space, we exploited the possibility of geometrically controlling $\tilde{\varepsilon }$ by just printing dielectric samples with different amounts of vacuum inside. The samples were printed with a high-temperature photopolymer material, using the PolyJet deposition technique. The high spatial resolution of the printer allowed the realization of artificially porous samples. The effective permittivity ${\tilde{\varepsilon }}_{eff}$ of the samples was controlled by printing empty columns across the whole sample thickness. The columns are arranged on square or hexagonal lattices: varying the lattice parameter $l_{p}$ and the column diameter $\emptyset_{h}$, samples with different filling percentages are obtained. The samples are electromagnetically homogeneous because $l_{p}\ll \lambda$ with λ the wavelength in the medium. At ∼13 GHz and with $\varepsilon^{\prime }∼3$ (anticipating the results shown in Figure 8), λ∼13 mm. A sketch of the samples is shown in Figure 9, and the characteristics of the samples are reported in Table 4.

**Figure 10:**
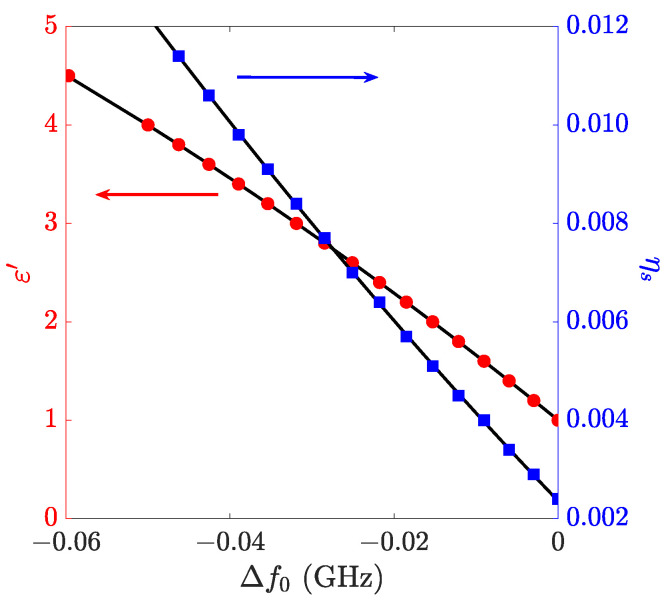
Calibration curves $\varepsilon^{\prime }\left(\Delta f_{0}\right)$ (red circles) and $\eta_{s}\left(\Delta f_{0}\right)$ (blue squares). The points are obtained with e.m. simulations and fitted with a 2nd-order polynomial. The obtained calibration curves are reported in Equations (10) and (11).

The following Section 3.2 samples of nominal thickness t=1.75 mm were printed to match the maximum measurement sensitivity, considering the mounting of the sample on the mask with the 13 mm central hole. The variations in the resonance frequency $\Delta f_{0}=f_{0,s}-f_{0,ref}$ measured with or without a loaded sample, and considering air as a reference, are simulated for different $\varepsilon^{\prime }$ values and shown in Figure 10. Hence, the calibration curve $\varepsilon^{\prime }\left(\Delta f_{0}\right)$ is obtained.

(10) $$\varepsilon^{\prime }\left(\Delta f_{0}\right)=-151.7\left(1.6\right)\Delta f_{0}^{2}-67.50\left(8\right)\Delta f_{0}+1.0020\left(10\right)\;,$$

where the numbers in parentheses are the numerical values of the standard uncertainties referred to the corresponding last digits of the quoted parameters. For each of these points, the sample filling factor $\eta_{s}$ is computed (see Figure 10) and the corresponding calibration curve $\eta_{s}\left(\Delta f_{0}\right)$ obtained:

(11) $$\eta_{s}\left(\Delta f_{0}\right)=0.4620\left(5\right)\Delta f_{0}^{2}-0.17390\left(5\right)\Delta f_{0}+0.0023610\left(5\right)\;,$$

The simulated points used to obtain the 2nd-order polynomial calibration curves are shown in Figure 10.

So, once $\Delta f_{0}$ are measured, with Equations (10) and (11), $\varepsilon^{\prime }$ and $\eta_{s}$ of the sample can be obtained.

In order to measure the resonance frequency variation $\Delta f_{0}$ with respect to an air-reference sample, a ring of the same thickness as the samples is printed in order to compare $f_{0}$ and *Q*, keeping the upper metallic closing cap at the same height and thus not changing the geometry of the DR. This ring has a central circular hole with an inner diameter ∅=(14.00±0.01) mm, and thus larger than the mask hole, in order not to interfere with the e.m. magnetic field. $\Delta (Q^{-1})$ and $\Delta f_{0}$ are then determined by measuring *Q* and $f_{0}$ of the DR loaded with the full square samples and with the ring.

The measurement procedure and uncertainty evaluation are now described: The VNA IF bandwidth was set to 10 kHz and every point was averaged over five acquisitions. The frequency span was chosen as ∼5 FWHMs [43,44,45] of the resonance curve. For each sample, the mounting was repeated 10 times. All the measurements were performed after a 12-terms VNA calibration performed with SOLT standards. Each sample (including the reference rings) was loaded into the DR in place of the metallic sample and then covered with a brass cap. The thickness *t* of every sample was measured with a micrometer, and the standard deviation of 10 repeated measurements was used as the uncertainty u(t).

The $f_{0}$ standard deviation of the 10 different mountings is always $u_{m}\left(f_{0}\right)/f_{0}<10^{-6}$. Because in real cases the height of the DR changes when the reference or the sample is mounted, the uncertainty u(t) was taken into account and propagated on the overall $u(f_{0})$ through the sensitivity function $\partial f_{0}/\partial t$ evaluated with numerical simulations. Thus, $u^{2}\left(f_{0}\right)=u_{m}^{2}\left(f_{0}\right)+u_{t}^{2}\left(f_{0}\right)$ where $u_{t}\left(f_{0}\right)=u\left(t\right)\partial f_{0}/\partial t$. Then, $\Delta f_{0}=f_{0,R}-f_{0,S}$ between the samples (subscript *s*) and rings (subscript *R*) of the same nominal thickness is evaluated and the uncertainty is obtained with the standard propagation procedure $u^{2}\left(\Delta f_{0}\right)=u^{2}\left(f_{0,R}\right)+u^{2}\left(f_{0,s}\right)$.

Once $f_{0}$ and $u(f_{0})$ are correctly evaluated, these can be used to evaluate $\varepsilon^{\prime }$ and $u(\varepsilon^{\prime })$ with Equation (10) and η, u(η) with Equation (11). Finally, using Equation (5), $\tan\delta_{s}$, and hence $\varepsilon^{\prime \prime }$, and their uncertainties, can be evaluated. The results are reported in Figure 8.

The uncertainties on the filling percentage of the samples are obtained starting from the 3D printer spatial accuracy and then analytically propagated using the geometrical properties of the lattices shown in Table 4.

Both $\varepsilon^{\prime }$ and $\varepsilon^{\prime \prime }$ show a linear dependence on the filling percentage γ of the sample in agreement with the effective medium theory and the upper limit of the Wiener model [55,56] and other literature works [57,58,59].

The results are also in agreement with those shown in other works. In [60], acrylonitrile butadiene styrene (ABS) samples doped with different quantities of BaTiO_3_ microparticles were measured with a split-post resonator at 15 GHz, obtaining $2.6<\varepsilon^{\prime }<8.7$ and 0.005<tanδ<0.027, whereas a broadband characterization (from 1 MHz to 11 GHz) was performed in [61]. The high-frequency range (8.2÷11) GHz was analyzed with the Nicholson–Ross–Weir reflection method [61]. Even if no measurement uncertainties are indicated, the results show $2.50<\varepsilon^{\prime }<3.29$ and 0.005<tanδ<0.037.

Finally, an experimental validation of the measurement accuracy of the new DR configuration on $\varepsilon^{\prime }$ was shown in [62], comparing the $\varepsilon^{\prime }$ measurements obtained with this resonant set-up and with a transmission/reflection standard technique based on the so-called “NIST-precision” method [63], which is an improved version of the Nicholson–Ross–Weir, and using a WR90 waveguide. With this set-up, we obtained $\varepsilon^{\prime }∼3.1$ [62], which is fairly in agreement with the here shown value measured on the full sample $\varepsilon^{\prime }=2.9\left(1\right)$.

## 5. Comparison with the State of the Art

The measurement fixture and measurement methods shown in this paper are the results of careful work, carried out in the last years, oriented to the development of microwave measurement techniques and systems for the characterization of materials (both conductors and dielectrics). In particular, and differently from many other works, we put a strong focus on the analysis and improvement in the metrological characteristics. In fact, it is clear how materials characterization methods at microwave frequencies were an evolving field in the last years: these techniques had great success in the experimental investigation of the e.m. properties of matter [64,65,66,67,68], and nowadays, microwave measurements are instrumental both for the design and testing of devices and complex systems whose performances can be particularly sensitive to the so-measured quantities [24,69,70,71,72,73,74,75,76] and in metrology [77,78,79]. This is the general framework into which this work fits: it shows a novel configuration of a dielectric-loaded resonator, and its possible applications, with a thorough evaluation of the measurement uncertainty.

Because one of the most detrimental sources of uncertainty in microwave resonant systems is the generally poor mounting repeatability, the presented fixture was specifically thought to overcome this point. Thus, a good figure of merit for this comparison can be the uncertainty u(Q) on the quality factor measurement. From the repetitions shown in Figure 4, we assessed that the standard relative uncertainty on the *Q* measurements for this fixture is u(Q)/Q=0.1%, including also the mounting repeatability. This can be compared with the “few” percent declared in [25], the 1% in [80], or the 4% in the international standard [81], even if in all these works no information about the repeatability is provided. We can conclude that with this new design, we improved the uncertainty on the *Q*-factor measurement of about one order of magnitude with respect to other literature works.

The complete description of the resonator and of its metrological characteristics here reported were not presented elsewhere, although some potential of the fixture was anticipated. Some preliminary results, based on differential surface resistance measurements, were previously reported in [82]. We recently proposed the use of this DR as a contactless surface roughness measurement fixture [83], taking advantage of the high measurement repeatability. For what concerns its use for the characterization of dielectric materials, we previously showed a preliminary optimization of the fixture in [62], which was then adapted to the configuration here presented, that was more extensively tested in this paper.

A discussion of the general metrological performances on the measurements of the physical quantities reported in this paper is in order. The relative measurement uncertainties $u\left(\varepsilon^{\prime }\right)/\varepsilon^{\prime }∼3\%$ and u(tanδ)/tanδ∼10% obtained with this resonator are aligned with other works: $u\left(\varepsilon^{\prime }\right)/\varepsilon^{\prime }∼1\%$ and u(tanδ)/tanδ∼20% are declared in [84] on ABS samples at 10 GHz, $u\left(\varepsilon^{\prime }\right)/\varepsilon^{\prime }∼1\%$ and u(tanδ)/tanδ∼0.2% (but simply and incompletely evaluated as the standard deviation of six measurements) on doped ABS samples in [60], and $u\left(\varepsilon^{\prime }\right)/\varepsilon^{\prime }\leq 5.8\%$ and 10%<u(tanδ)/tanδ∼0.2%<200% on 3D printed ABS with the Nicholson–Ross–Weir waveguide method in [61]. Looking at measurements of $\tilde{\varepsilon }$ only, the method here presented does not significantly improve the uncertainties with respect to other methods. However, the novelty of this method is still of interest in several applications due to the ease of use and to the possibility of characterizing with the same fixture both dielectric materials (even in the presence of back metal plates) and conductors. A planned application of this system is the measurement of the surface impedance/complex permittivity of bad conductors, such as conductive paints or a semiconductor.

Finally, it is worth noting that the uncertainties shown in the previous sections also take advantage of the optimization on the *Q* and $f_{0}$ measurement procedure and algorithm, based on the complex Lorentzian fit of the transmission *S*-parameters, that we extensively discussed in [43].

## 6. Summary

Due to the rapid spread of additive manufacturing techniques, and also, for perspective, high-frequencies applications, the interest in microwave measurement systems able to characterize the e.m. properties of these materials is increasing. For this reason, we designed a new measurement fixture, based on a dielectric-loaded resonator DR, able to measure either the surface resistance $R_{s}$ of conductive samples or the complex permittivity $\tilde{\varepsilon }$ of dielectric samples. The DR was designed to obtain a measurement system that is easy to use and versatile enough to quickly test the properties of different materials used in prototyping laboratories: in the proposed configuration, the samples can be loaded from the outside of the resonant cell without the need to disassemble the whole resonator for each measurement. This feature also allowed to enhance the typical low measurement repeatability provided by this kind of fixture.

In this paper, the optimization procedure followed to maximize the DR measurement sensitivity is discussed and the final geometry is reported. The designed DR was then realized and its metrological characteristics, in terms of the measurement repeatability and uncertainties, were carefully evaluated. The relative standard deviations of the *Q* and $f_{0}$ measurements obtained in 20 repeated mountings are $10^{-3}$ and $15\times 10^{-6}$, respectively. These are about one order of magnitude smaller than the typical values obtainable with this kind of fixture. The realized DR was then experimentally tested on the measurement of $R_{s}$ of different conductive samples and on the $\tilde{\varepsilon }$ measurement of dielectric samples. In particular, to highlight the performances of the designed fixture in terms of the effect of the excellent measurement repeatability, the DR was first used for the measurement of the $R_{s}$ variation among different samples, using one of these as the reference. This approach allowed us to evaluate the uncertainty $u(\Delta R_{s})=1.1\;m\Omega$ which is given only by the uncertainties u(Q) and u(G). In the text, a procedure for an in situ calibration of the resonator is shown to obtain absolute $R_{s}$ measurements. With this procedure, a relative standard uncertainty of 20% was obtained on copper. This highlights the importance that an $R_{s}$ standard would have in microwave measurements.

For what concerns the characterization of dielectric materials, the use of the designed DR for $\tilde{\varepsilon }$ measurements of 3D printing materials was shown. Due to the large variability that the loss tangent $\tan\delta_{s}$ can have among different materials, here an optimization of the dimensions of the sample to be measured must be performed, starting from an a priori estimation of $\tilde{\varepsilon }$ (if necessary, this optimization can be refined in successive steps). In this paper, the complex permittivity $\tilde{\varepsilon }$ of 3D printed samples with different porosity was measured. The linear dependence of both the real and imaginary parts of $\tilde{\varepsilon }$ found on the filling amount of the samples was shown to be in good agreement with the theoretical models. Thus, in addition to the good agreement between the obtained measurements and the literature values, that confirms the accuracy of the developed system also in the $\tilde{\varepsilon }$ measurements.

In summary, we developed a versatile microwave measurement fixture for the characterization of conductive and dielectric materials. The measurement uncertainties provided, the simple conceptual approach, and the ease of use make this fixture of potential interest for test laboratories involved in the design and prototype of microwave components and systems.

## Figures and Tables

**Table 1:** List of the measured sample. The nominal *R* is evaluated at ∼12.9 GHz from the dc resistivity ρ of the material, assuming $R_{s}=\sqrt{\mu_{0}\pi f\rho }$. The samples are square in shape and of dimensions $15\times 15\times 3\;{\mathrm{mm}}^{3}$.

Ref.	Material	Nominal *R* (mΩ)
R0	Brass	55÷68
R1	Copper	29
R2	Aluminum	38
R3	Zinc	55

**Table 2:** $\Delta R_{s}$ measurement results.

Sample	ΔR (mΩ)	u(ΔR) (mΩ)
R0	35.4	1.1
R1	ref	-
R2	11.0	1.1
R3	35.0	1.1

**Table 3:** Measurement results.

Sample	$R_{\mathrm{SM}}$ (mΩ)	u(R) (mΩ)
R0	92	12
R1	58	12
R2	68	12
R3	92	12

**Table 4:** Lattice characteristics of the samples.

Lattice Type	$l_{p}$ (mm)	$\emptyset_{h}$ (mm)	Filling %
-	-	-	100
square	1.60	0.40	81
hexagonal	1.00	0.30	68
hexagonal	1.50	0.56	50

## Data Availability

The data are available on request to the corresponding author.
